# MD simulations explain the excess molar enthalpies in pseudo-binary mixtures of a choline chloride-based deep eutectic solvent with water or methanol

**DOI:** 10.3389/fchem.2022.983281

**Published:** 2022-11-14

**Authors:** Leon de Villiers Engelbrecht, Xiaoyan Ji, Carlo Maria Carbonaro, Aatto Laaksonen, Francesca Mocci

**Affiliations:** ^1^ Department of Chemical and Geological Sciences, University of Cagliari, Cagliari, Italy; ^2^ Division of Energy Science, Energy Engineering, Luleå University of Technology, Luleå, Sweden; ^3^ Department of Physics, University of Cagliari, Cagliari, Italy; ^4^ Division of Physical Chemistry, Arrhenius Laboratory, Department of Materials and Environmental Chemistry, Stockholm University, Stockholm, Sweden; ^5^ Center of Advanced Research in Bionanoconjugates and Biopolymers, “Petru Poni” Institute of Macromolecular Chemistry, Iasi, Romania; ^6^ State Key Laboratory of Materials-Oriented and Chemical Engineering, Nanjing Tech University, Nanjing, China

**Keywords:** deep eutectic solvent, choline chloride, cosolvents, excess properties, pseudo-binary solvent mixture, MD simulations

## Abstract

The addition of molecular liquid cosolvents to choline chloride (ChCl)-based deep eutectic solvents (DESs) is increasingly investigated for reducing the inherently high bulk viscosities of the latter, which represent a major obstacle for potential industrial applications. The molar enthalpy of mixing, often referred to as excess molar enthalpy *H* ^E^—a property reflecting changes in intermolecular interactions upon mixing—of the well-known ChCl/ethylene glycol (1:2 molar ratio) DES mixed with either water or methanol was recently found to be of opposite sign at 308.15 K: Mixing of the DES with water is strongly exothermic, while methanol mixtures are endothermic over the entire mixture composition range. Knowledge of molecular-level liquid structural changes in the DES following cosolvent addition is expected to be important when selecting such “pseudo-binary” mixtures for specific applications, e.g., solvents. With the aim of understanding the reason for the different behavior of selected DES/water or methanol mixtures, we performed classical MD computer simulations to study the changes in intermolecular interactions thought to be responsible for the observed *H* ^E^ sign difference. Excess molar enthalpies computed from our simulations reproduce, for the first time, the experimental sign difference and composition dependence of the property. We performed a structural analysis of simulation configurations, revealing an intriguing difference in the interaction modes of the two cosolvents with the DES chloride anion: water molecules insert between neighboring chloride anions, forming ionic hydrogen-bonded bridges that draw the anions closer, whereas dilution of the DES with methanol results in increased interionic separation. Moreover, the simulated DES/water mixtures were found to contain extended hydrogen-bonded structures containing water-bridged chloride pair arrangements, the presence of which may have important implications for solvent applications.

## 1 Introduction

The deep eutectic solvents (DESs) are a diverse group of eutectic mixtures of two or more components, i.e., they have a lower melting point (mp) than expected for a corresponding ideal mixture of the components, and they exhibit a characteristic mp minimum, i.e., the eutectic point. In fact, the “DES” assignment implies that the mixture composition is close to, or at, this eutectic point (Smith et al., 2014). DESs are often composed of an ionic hydrogen bond acceptor (HBA), e.g., quaternary ammonium halide salt, and a metal salt or an electronically neutral hydrogen bond donor (HBD). The choline chloride (ChCl)-based DESs, involving a neutral HBD, typically urea or ethylene glycol, glycerol, or malonic acid, exhibit particularly favorable properties as alternative solvents, and are actively studied (Hansen et al., 2021); in fact, these so-called “type III” DESs have even been assigned unique names, e.g., the ChCl/urea 1:2 molar ratio mixture is widely referred to as “reline”, the corresponding mixture with ethylene glycol as “ethaline”, and so on. In these systems, the HBD is thought to strongly coordinate, or complex the chloride anion, reducing the strength of its electrostatic interactions with the organic cation, and thus the mp (Abbott et al., 2003; Ashworth et al., 2016).

DESs share many favourable solvent properties with the related ionic liquids (ILs), e.g., low vapor pressure, wide liquid temperature range, and low flammability, and they are often considered IL analogues (Ma et al., 2018). The ChCl-based DESs, in particular, exhibit a number of additionally advantageous properties compared to established ILs, which include facile synthesis from readily available, inexpensive, and environmentally benign starting materials under moderate conditions (Zhang et al., 2012). As for ILs, however, the typically high viscosities of these DESs under ambient conditions represent a major obstacle to their applications as solvents or lubricants (García et al., 2015; Ma et al., 2018). The addition of more highly mobile molecular cosolvents, including water or alcohols, to form a “pseudo-binary” (since the DES itself is a mixture) liquid mixture has been proposed as a possible solution to this problem (Xie et al., 2014; Yadav et al., 2014; Yadav and Pandey, 2014; Harifi-Mood and Buchner, 2017); moreover, many DESs (like ILs) are hygroscopic, readily absorbing atmospheric water. While the addition of cosolvents to DESs has been shown to effectively reduce their viscosity and modify other macroscopic liquid properties, e.g., density, and it is also expected to affect the characteristic intermolecular interactions and molecular arrangements within the DESs, which may have important implications for their solvent characteristics.

Information regarding changes in intermolecular interactions and arrangements that occur when two liquids are mixed may be obtained through the measurement of excess thermodynamic properties, e.g., the excess molar enthalpy (*H* ^E^), and/or excess molar volume (*V* ^E^). DES/cosolvent mixtures have been found to exhibit significantly nonideal mixing behavior, often showing interesting, unexpected excess thermodynamic property variations as a function of cosolvent content (Ma et al., 2018). These include the sinusoidal (S-shaped) *H* ^E^ trends of aqueous ChCl/urea DES mixtures as a function of water content, in which initially negative *H* ^E^ (exothermic mixing, i.e., strong intermolecular interactions between DES components and water) changes to positive (endothermic) with increasing water content. Wang et al. (Wang Y. et al., 2020), in a recent thermodynamics study of mixtures of ethaline or the corresponding ChCl/glycerol 1:2 molar ratio DES (“glyceline”) with either water or methanol (at 308.15 and 318.15 K), found the *H* ^E^ of aqueous mixtures to be strongly negative, while those of methanol mixtures were small positive values at all mixture compositions. While addition both water and methanol reduce the DES viscosity, the opposite sign of the resulting *H* ^E^ point to different intermolecular interaction changes upon mixing.

The ChCl-based DESs themselves are characterized by considerable microstructural complexity (Spittle et al., 2022), in which different interaction modes among components are possible; in fact, these DESs have been described as an “alphabet soup” of hydrogen bonding (“H-bonding”) interactions (Ashworth et al., 2016). Computational techniques, and classical Molecular Dynamics (MD) simulations in particular, have been extensively employed to study the solvation of simple or complex solutes (Laaksonen et al., 2012; Engelbrecht et al., 2018), the structural organization in liquids and ILs (Wang Y.-L. et al., 2020; Engelbrecht et al., 2022), and to rationalize their experimentally observed properties (Mariani et al., 2017; Demurtas et al., 2021; Lengvinaitė et al., 2021). These computational methods are also increasingly used to study DESs and DES-containing systems (Velez and Acevedo, 2022), often in combination with experimental methods (Hansen et al., 2021; Spittle et al., 2022). Several MD simulation studies of DES/water pseudo-binary mixtures, which naturally are highly complex, have been reported in recent years (Gao et al., 2018; Kumari et al., 2018; Baz et al., 2019; Celebi et al., 2019; Kaur et al., 2020). The present work describes an MD simulation study of ethaline/water and ethaline/methanol mixtures, with an emphasis on first reproducing, and then rationalizing their striking experimental *H* ^E^ sign difference (Wang Y. et al., 2020).

## 2 Methods

### 2.1 Force field

The General Amber Force Field (GAFF) was used to model the ChCl/ethylene glycol DES and its mixtures with methanol (Wang et al., 2004). The DES partial atomic charges of Perkins et al. (Perkins et al., 2014), developed specifically for use with GAFF parameters, were used (with ionic species charges scaled by a factor of 0.9). For methanol, an established set of partial atomic charges were obtained from the online R.E.D database, developed using the GAFF-consistent RESP (HF/6-31*) fitting procedure (Dupradeau, 2005; Dupradeau et al., 2010); the SPC/E model was used for water (Berendsen et al., 1987).

### 2.2 Starting configurations

Starting configurations were prepared using the Packmol software (Martínez et al., 2009), randomly placing a total of 300 choline (Ch^+^) and chloride (Cl^−^) ions, and 600 ethylene glycol (EG) molecules in a periodic cubic simulation cell, with dimensions chosen such that the configuration is close to target density. For DES/cosolvent mixtures, appropriate numbers of DES components and cosolvent molecules were placed in cubic cells of similarly chosen dimensions, detailed in Table 1. Here, the DES/cosolvent composition is expressed as a cosolvent mole fraction, *x*_cosolvent_, which is computed as follows,

$$x_{cosolvent}=\frac{N_{cosolvent}}{N_{ChCl}+N_{HBD}+N_{cosolvent}}$$ (1)

where *N*_ChCl_ is the number of ChCl ion pairs, *N*_HBD_ is the number of HBD molecules, and *N*_cosolvent_ is the number of cosolvent molecules.

**TABLE 1:** Details of simulated DES/cosolvent mixtures and respective pure solvents. The total number (*N*) of units/molecules of each component (choline chloride ChCl, ethylene glycol EG, and water/methanol cosolvent) and the simulation average densities (308 K, 0.98 bar) are shown.

System	*x*_cosolvent_	*N*_ChCl_	*N*_EG_	*N*_cosolvent_	Density (g.cm^−3^)
Pure DES	—	300	600	—	1.1044
DES/water	0.125	300	600	129	1.1041
	0.250	300	600	300	1.1029
	0.500	300	600	900	1.0953
	0.750	300	600	2,700	1.0708
	0.875	150	300	3,150	1.0430
	1.000	—	—	3,000	0.9902
DES/methanol	0.125	300	600	129	1.0886
	0.250	300	600	300	1.0695
	0.500	300	600	900	1.0195
	0.750	150	300	1,350	0.9400
	0.875	75	150	1,575	0.8804
	1.000	—	—	1,000	0.7976

### 2.3 Simulation methods

All MD simulations were performed using the Amber 16 software package (Case et al., 2016). The simulation procedure was essentially similar to that described by Perkins et al. (Perkins et al., 2014): following system equilibration (see below), NPT simulations (308 K, 0.98 bar) were performed using Langevin dynamics (5 ps^−1^ time constant), with weak (Berendsen et al., 1984) pressure coupling (1 ps time constant), and a 2 fs simulation time step; bonds involving hydrogen atoms were constrained using the SHAKE algorithm (Ryckaert et al., 1977). The nonbonded interaction cutoff was set at 15 Å, with long-range electrostatic interactions computed using the PME procedure (Darden et al., 1993), tolerance 10^−5^. The equilibration of the system was performed through a combination of steepest-descent energy minimization and NPT MD simulations; a detailed description of these steps is given in the Supplementary Material. After the equilibration phase, 20 ns long NPT production simulation trajectories were generated for each system.

## 3 Results

### 3.1 Excess molar enthalpies

**FIGURE 1:**
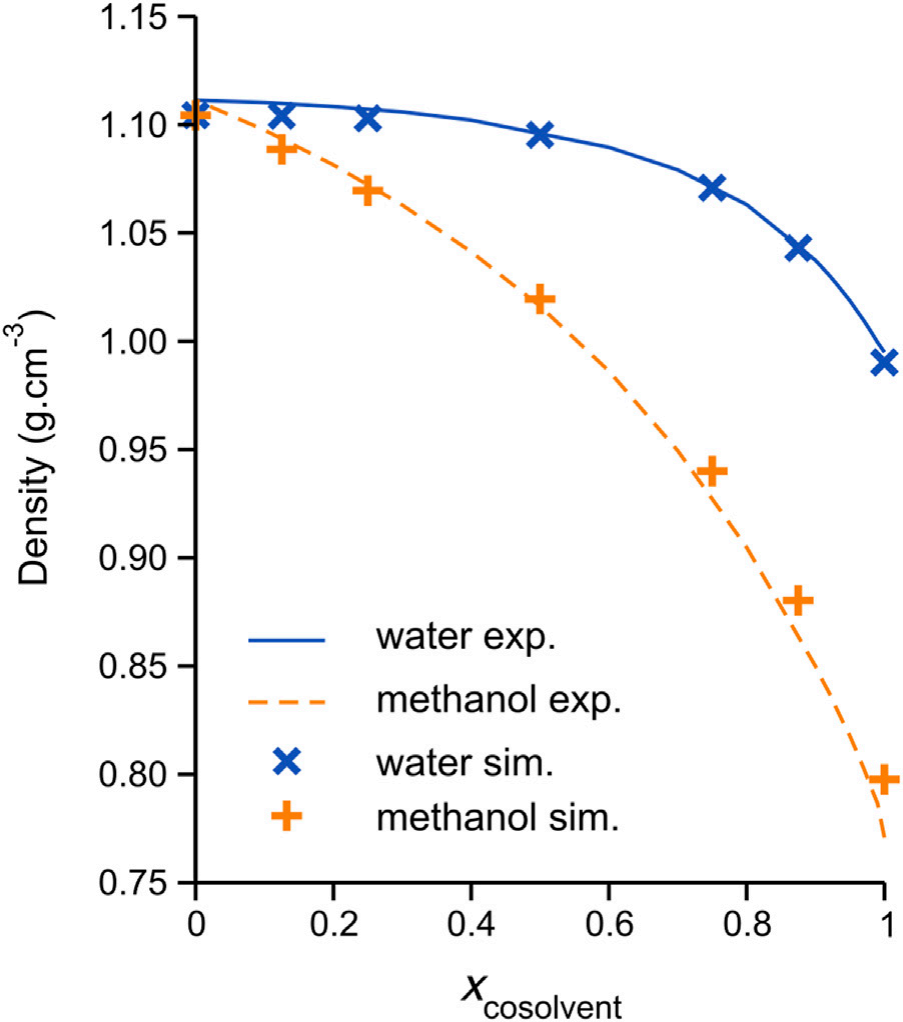
Average densities of simulated ChCl/ethylene glycol (1:2 molar ratio) DES and pseudo-binary mixtures with either water (blue crosses) or methanol (orange plus signs) cosolvent at 308.15 K. The solid blue and dashed orange lines connect the recently reported experimental data of Wang et al. (Wang Y. et al., 2020) for the water and methanol mixtures, respectively. The density data is plotted as a function of mixture composition, expressed as a mole fraction cosolvent.

The accurate reproduction of experimental bulk liquid densities constitutes an important target in the validation of DES models (as for many other liquid systems); in fact, the current DES model ionic charges, originally developed by Perkins et al. (Perkins et al., 2014), were scaled by the authors (by a factor 0.9) to better reproduce experimental densities and transport properties compared to the corresponding model with full ionic charges (±1 e). Scaling of the ionic solute charges has been used in many previous investigations to account for the charge transfer phenomena; see Engelbrecht et al. (Engelbrecht et al., 2018) and references therein. The calculated average densities of all simulated DES/cosolvent systems are plotted in Figure 1, along with the recently reported experimental measurements of Wang et al. (Wang Y. et al., 2020); corresponding numerical data are reported in Table 1. The simulation average values may be seen to be in generally good agreement with the experimental data, with the methanol mixtures showing increasing deviation at higher methanol content; in fact, the average density of the pure methanol model is noticeably higher than the experiment; 0.797 and 0.771 g/cm^3^, respectively. The discrepancies between simulated and experimental densities are of effectively comparable magnitude to those previously reported for classical MD simulations of these systems, specifically by Kaur et al. (Kaur et al., 2020) for the DES/water mixtures, using a refined CHARMM-based DES force field (Kaur et al., 2019) with SPC/E water (Berendsen et al., 1987), and, very recently, by Cea-Klapp et al. (Cea-Klapp et al., 2022) for the methanol mixtures, using the OPLS-AA-based (“OPLS-DES”) force field of Doherty and Acevedo (Doherty and Acevedo, 2018). Notably, the DES/methanol simulations of Cea-Klapp et al. more accurately reproduce the mixture density at high methanol content, which is not surprising considering the employed OPLS-AA methanol model was parameterized to reproduce bulk liquid properties (Jorgensen and Tirado-Rives, 1996).

The excess molar enthalpy, *H* ^E^, may be computed from computer simulations as follows (Dai et al., 2010),

$$H^{E}=U_{m}-\sum x_{i}U_{i}+PV^{E}$$ (2)

where *U*_m_ is the average potential energy per molecule in the mixture, *U*_*i*_ is the corresponding values for the respective pure liquids, and *x*_*i*_ is their mole fractions in the mixture; *p* is the pressure, and *V* ^E^ is the excess molar volume of the mixture. Since the *V* ^E^ of liquid mixtures are small (Chitra and Smith, 2001), the *PV* ^E^ term is often omitted from the calculation, in which case *H* ^E^ is approximated as the excess molar potential energy, *U* ^E^ (Walser et al., 2000; Wu et al., 2005):

$$H^{E}\approx U^{E}=U_{m}-\sum x_{i}U_{i}$$ (3)

The calculation of *H* ^E^ from computer simulations of ChCl-based DES/cosolvent mixtures presents somewhat of a conceptual problem, since the DES component itself is a mixture containing a dissociable ionic component (ChCl); in fact, to the best of our knowledge, no computer simulation studies of the excess molar enthalpies of DES/cosolvent mixtures have been reported to date. Baz et al. (Baz et al., 2019), in their computational investigation of the thermophysical properties of glyceline/water mixtures (including *V* ^E^ and water activity coefficients), proposed an alternative mole fraction definition as appropriate for simulated DES/cosolvent systems, which considers the dissociated ChCl ion pair (as opposed to the more widely used definition in Eq. 1):

$$x_{cosolvent}^{species}=\frac{N_{cosolvent}}{N_{{Ch}^{+}}+N_{{Cl}^{-}}+N_{HBD}+N_{cosolvent}}$$ (4)

In the present work, the *H* ^E^ results of the simulated systems were computed using Eq. 3, with average potential energies computed over the last 10 ns of each simulation. The statistical uncertainties in the computed data were evaluated by a block averaging procedure (Flyvbjerg and Petersen, 1989). In view of the established practice of computing *H* ^E^ of simulated IL/cosolvent mixtures using mole fractions that consider IL ion pairs, e.g. (Wu et al., 2005), as well as considerations presented in a recent computational study of the vapor pressures of ChCl-based DESs (Salehi et al., 2021), including ethaline, the more common DES/cosolvent mole fraction definition in Eq. 1 was employed in the calculations. For comparison, we repeated the *H* ^E^ calculations using the alternative mole fraction (species cosolvent) definition in Eq. 4, resulting in qualitatively similar trends (see below).

**FIGURE 2:**
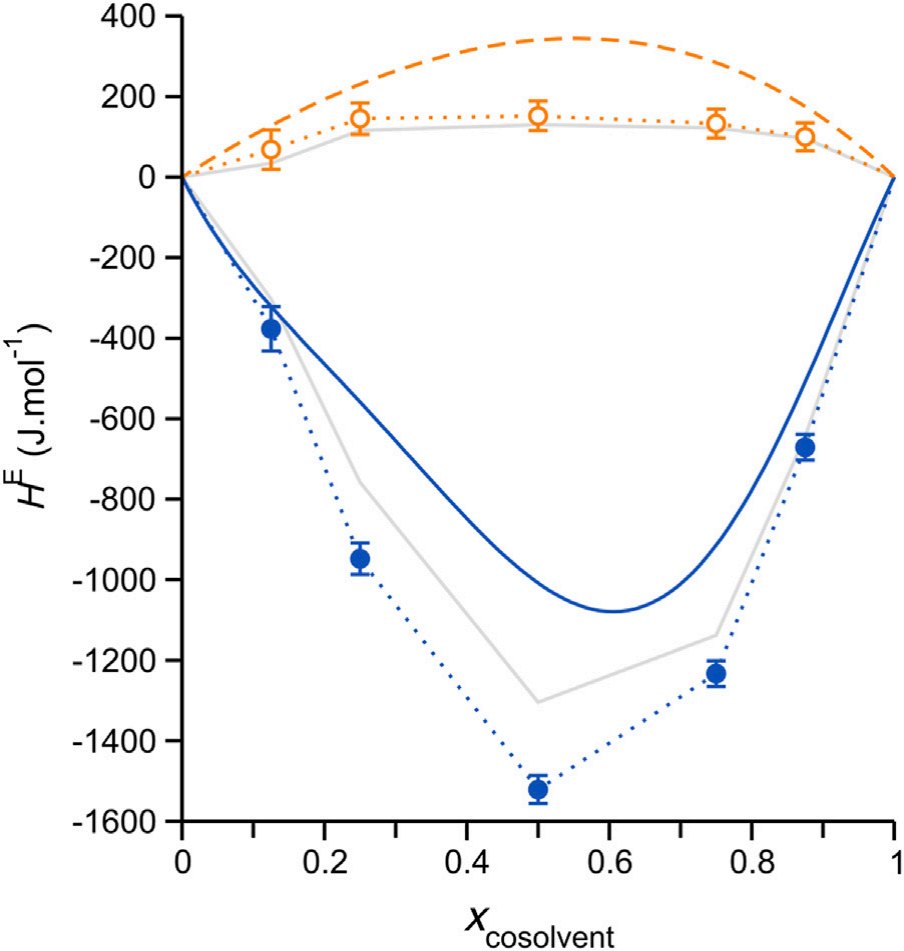
Excess molar enthalpies, *H* ^E^, computed for simulated ChCl/ethylene glycol (1:2 molar ratio) DES mixtures with either water (blue solid circle markers) or methanol (open orange circles) cosolvent at 308.15 K, plotted as a function of the mole fraction cosolvent. Dotted lines of corresponding color connect the data, serving as guides to the eye; error bars represent one standard deviation. The smooth solid blue and dashed orange lines are non-random two liquid (NRTL) model fits to the experimental *H* ^E^ data reported by Ji and coworkers (Ma et al., 2016; Ma et al., 2017; Wang Y. et al., 2020). The experimental errors for the mixtures with water are 5–10% (Ma et al., 2016) and those for the mixtures with methanol are a bit higher depending on the absolute value, with maximum errors up to 15% (Wang Y. et al., 2020). Solid grey lines without data markers show the *H* ^E^ computed using an alternative component mole fraction definition, which considers the dissociated ChCl ion pair, as discussed in the main text; error bars have also been omitted for these data in the interest of clarity (similar in magnitude to those associated with the circle-marked data).

The *H* ^E^ results obtained from our simulations are plotted in Figure 2, where they are compared with the experimental data of Wang et al. (Wang Y. et al., 2020). Despite substantial discrepancies, the simulations effectively reproduce the experimental sign difference (aqueous mixtures, strongly negative; methanol mixtures, low positive values) and essential trend shapes. Concerning the actual magnitude of the simulation/experimental deviations in Figure 2, it should be noted that these are generally consistent with those previously reported for liquid mixtures modeled using classical force fields, e.g. (Dai et al., 2010), including IL mixtures (Wu et al., 2005). The *H* ^E^ values computed using the alternative mole fraction definition, Eq. 4, (solid grey lines with data markers omitted) are smaller in an absolute sense, substantially improving the agreement with the experiment for the aqueous mixtures. Finally, we note that the sign of deviations from the experiment are negative (i.e., smaller positive values for the DES/methanol systems, more negative values for the aqueous systems), which hints at an overestimation of the DES-cosolvent interaction strength in the simulations, likely electrostatic interactions (including H-bonding) (Engelbrecht et al., 2021). Nevertheless, despite uncertainty at this stage as to the more appropriate approach for computing *H* ^E^ for these systems, the simulations clearly reproduce the key differences and general trends found experimentally.

### 3.2 Liquid structure

#### 3.2.1 Radial distribution functions

The liquid structures of simulated DESs and their aqueous mixtures are routinely analyzed by radial distribution functions (RDFs), each describing the radially averaged correlation between a particle pair, and, together, representing the simulation average structure. These functions also have useful experimental counterparts, namely, neutron and X-ray scattering patterns, which have been reported for some ChCl-based DES systems (Hammond et al., 2016, 2017; Kumari et al., 2018; Kaur et al., 2019, 2020). While RDFs are widely used in the structural characterization of liquid systems (Engelbrecht et al., 2022), H-bonded liquids and mixtures in particular, the high structural complexity of DESs and their mixtures require the evaluation of a large number of such functions, and workers often resort to computing molecular “center-of-mass” (COM) RDFs in order to obtain more general information about structural rearrangements upon mixing (Kaur et al., 2017; Gao et al., 2018).

**FIGURE 3:**
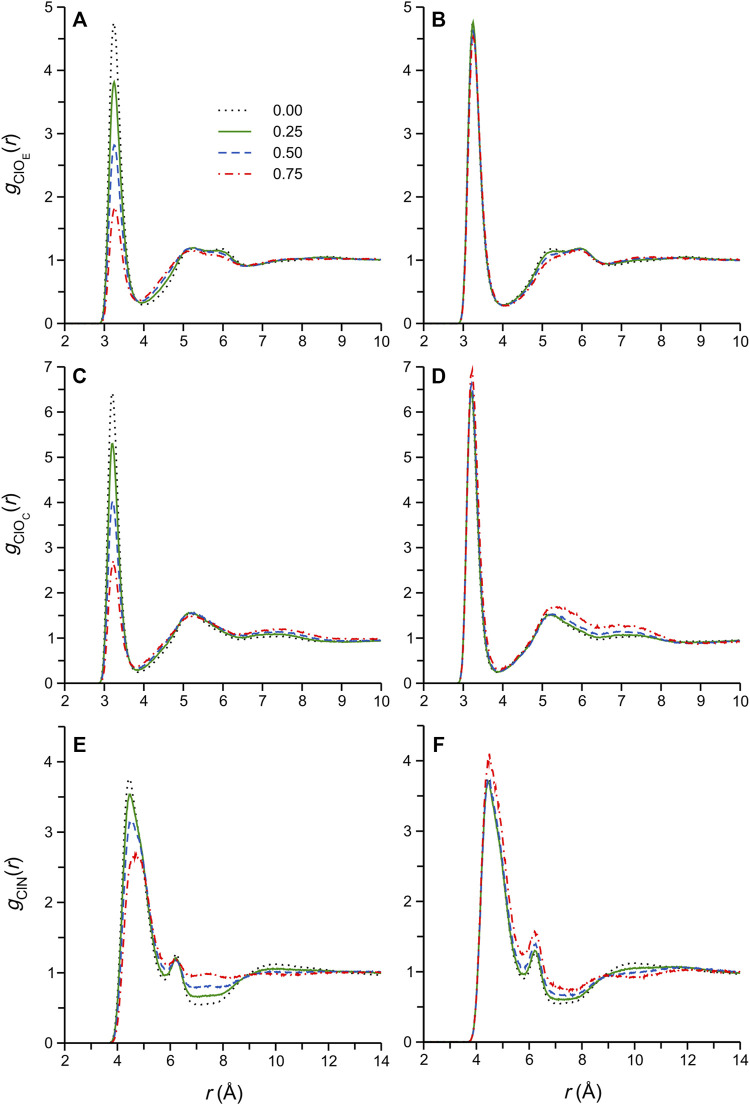
Radial distribution functions, *g*_Cl*X*_(*r*), computed for Cl^−^-*X* atom pairs for selected DES/water **(A,C,E)** and DES/methanol **(B,D,F)** systems, where *X* = O_E_ (EG oxygen atoms), O_C_ (cation oxygen atom), or N (cation N atom). The legend in **(A)** pertains to all other panels as well, and shows the mole fraction of the cosolvent in the corresponding simulated system.

Strong ionic H-bonding interactions involving the DES Cl^−^ anion are widely accepted to play a central role not only in DES formation (Abbott et al., 2003), during which the anion is thought to be effectively coordinated by the HBD (thus disrupting strong electrostatic interactions between the ions, and decreasing the mp) (Ashworth et al., 2016), but also in determining the excess thermodynamic properties upon mixing of the DES with a cosolvent (Gao et al., 2018). In fact, a recent MD simulation study of aqueous DES mixtures, including ethaline, highlighted the critical role of Cl^−^ in determining the physical properties of these DES solutions (Celebi et al., 2019). In the present work, we start by considering Cl-*X* RDFs, *g*_Cl*X*_(*r*), where the *X* are H-bond donor atoms of the DES and cosolvent. The RDFs for the pure DES and selected cosolvent mixtures are shown in Figure 3 (water mixtures data on the left, methanol mixtures on the right).

The RDFs for the pure DES and its aqueous mixtures (Figures 3A,C,E) have been reported under different, though similar, physical conditions, and analyzed in some detail (Perkins et al., 2014; Ferreira et al., 2016; Celebi et al., 2019; Kaur et al., 2019). In these previous reports, the sharp first (closest) maxima, or peak, of the *g*_ClO_(*r*) functions, Figures 3A,C, is identified with H-bonding interactions (Cl^−^ ∙∙∙ H‒O) of Cl^−^ with EG and Ch^+^, respectively. The intensity of these well-defined maxima, occurring at distances *r*_ClO_ = 3.3–3.2 Å, decreases with increasing water content, reflecting the entry of water molecules into the Cl^−^ coordination shell, where they displace DES components (Celebi et al., 2019). The *g*_ClN_(*r*) results in Figure 3E, reporting spatial correlations between Cl^−^ and the Ch^+^ N atom, exhibit broad first maxima at greater distances, *r*_ClN_ > 4 Å, that result from electrostatic interactions between the anion and the bulky Ch^+^ ammonium group. At all aqueous mixture compositions, the second maximum of this function, occurring at *r*_ClN_ ≈ 6 Å, arises from Ch^+^ cations interacting with Cl^−^ *via* so-called “doubly ionic” H-bonds, i.e., Ch^+^ with relative orientations in which the -OH group is directed at Cl^−^. In the pure DES, the *g*_ClN_(*r*) first maximum has a noticeably asymmetric shape, suggesting the simultaneous presence of various (similarly favorable) interaction configurations, or modes, between Cl^−^ and the Ch^+^ ammonium group (Zahn et al., 2016; Stefanovic et al., 2017). Indeed, a number of different interaction modes were demonstrated by Ashworth et al. (Ashworth et al., 2016), and these are likely characterized by slightly different average Cl-N distances. The shape of the *g*_ClN_(*r*) first maximum gradually changes with increasing water content, with the position of the actual maximum shifting from ∼4.4 to 4.7 Å, suggesting a change in the predominant Cl^−^-Ch^+^ interaction mode upon dilution with water; this subtle change is also evident in the RDFs recently reported by Celebi et al. (Celebi et al., 2019) and Kaur et al. (Kaur et al., 2020).

Next, we consider the Cl-*X* RDFs of selected DES/methanol mixtures in Figures 3B,D,F, and note that these appear essentially invariant with respect to methanol content (compared to the corresponding functions for the aqueous mixtures), with first maximum intensities increasing slightly, and more noticeable changes only appearing at higher *x*_M_, particularly at *r*_Cl*X*_ > 4 Å. Interestingly, the asymmetric *g*_ClN_(*r*) first peak profile of the pure DES largely persists in the methanol mixtures, even at high dilution, suggesting that the characteristic native DES Cl^−^-Ch^+^ interaction mode distribution does not change appreciably upon the addition of methanol. These observations, which are in stark contrast to those described for the aqueous mixtures, are essentially consistent with the conclusions of Cea-Klapp et al. (Cea-Klapp et al., 2022), who very recently reported an MD simulation study of ethaline/methanol mixtures (to the best of our knowledge, the only other computational study of this system): “In DESs plus methanol mixtures, the basic characteristics of pure DESs are conserved. Despite this, adding methanol changes the structure of solvation shells, where the interaction of HBD with methanol takes centre stage”. The effectively constant RDF first maximum intensities with respect to methanol content in Figures 3B,D,E does not necessarily imply a more stable Cl^−^ coordination shell composition upon dilution with methanol as opposed to water, since the RDF intensities are normalized to the bulk particle density of the system, which changes with mixture composition; more complete information on the solvation shell composition may be obtained by appropriate RDF integration to yield coordination numbers (see below). Nevertheless, inspection of the RDFs in Figure 3 has provided interesting clues as to the different configurational changes in the Cl^−^ coordination shell region between the aqueous and methanolic DES mixtures.

**FIGURE 4:**
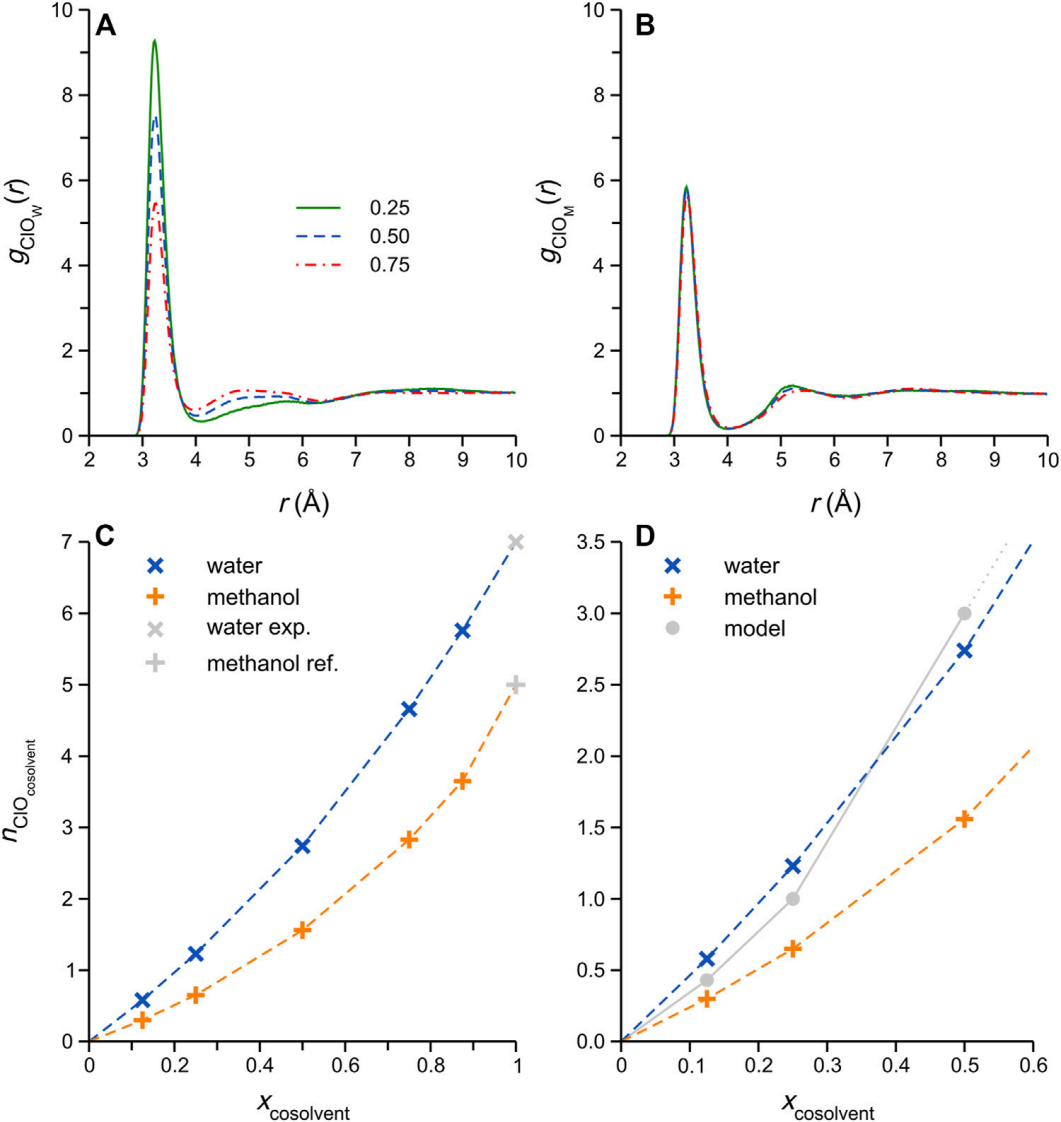
Cl^−^-O_cosolvent_ RDFs for selected **(A)** DES/water, and **(B)** DES/methanol simulated mixtures; the legend in panel A refers to the cosolvent mole fraction, and applies to both **(A,B)**. **(C)** Cl^−^-O_cosolvent_ coordination numbers, computed by integration of corresponding *g*_ClO_(*r*) first maxima, plotted as a function of cosolvent content. The Cl^−^ coordination number in the respective cosolvents (i.e. effectively infinite dilution) was taken from literature sources: Powell et al. (Powell et al., 1993) for the coordination number in water, and Impey et al. (Impey et al., 1987) for methanol. **(D)** Expansion of the low *x*_cosolvent_ range in **(C)**, showing the expected Cl^−^-cosolvent coordination number variation considering a simple strong Cl^−^-cosolvent attraction model (grey circles connected by solid grey line) described in the main text.

In order to obtain direct information about DES-cosolvent interactions in these systems, the Cl-water oxygen (O_W_) and -methanol oxygen (O_M_) RDFs were computed; these functions relate to ionic H-bonding interactions between Cl^−^ and the respective cosolvents, and selected examples are shown in Figures 4A,B. As noted in Figure 3, the water mixtures show significant changes in the Cl^−^-O_W_ RDFs with increasing cosolvent content, while the Cl^−^-O_M_ RDFs remain more-or-less unchanged. The relative intensities of the first (*r*_ClO_ ≈ 3.5 Å) and broader second maxima (*r*_ClO_ ≈ 4–6 Å) in the RDFs of the aqueous systems show an interesting change with increasing water content, suggestive of changes in the mixture microstructure, specifically water self-association (Kaur et al., 2020). Such changes are not observed in the Cl-O_M_ RDFs.

Cl^−^-cosolvent H-bonded coordination numbers, *n*_ClO_, were obtained by integration of the corresponding Cl^−^-O_W/M_ RDF first maxima (i.e., taking the integral up to the first local minimum). The results of this procedure, plotted in Figure 4C, reveal that, despite the very noticeable differences in the RDF structural changes between the water and methanol systems (Figures 4A,B), the respective coordination numbers exhibit rather similar variations as a function of cosolvent content. Note that the Cl^−^-cosolvent coordination numbers at effectively infinite dilution, i.e., *x*_cosolvent_ = 1, were taken from literature (Impey et al., 1987; Powell et al., 1993); interestingly, experimental reports of the coordination numbers of Cl^−^ in methanol obtained by neutron and X-ray scattering methods vary considerably (Faralli et al., 2006), between ca. 3.5 (Yamagami et al., 1995) and 6.2 (Megyes et al., 2002), and thus it was decided to adopt an intermediate value (5) derived from the computer simulation (Impey et al., 1987). For both mixtures, the *n*_ClO_ data follow curved trends with increasing cosolvent content, with the water coordination numbers consistently higher. The latter observation is not surprising, considering the higher polarity (partial atomic charges) and smaller size of water. However, closer inspection of the *n*_ClO_ data at low cosolvent content (*x*_W/M_ < 0.5) reveals that the Cl^−^-water coordination numbers are, indeed, unexpectedly high: consider a simple model in which the Cl^−^-cosolvent attraction is sufficiently strong (i.e., a sort of “strong-limit” scenario) that each cosolvent molecule added to the DES binds (*via* ionic H-bonding) to a single Cl^−^ anion, displacing the required number of DES components in the Cl^−^ coordination shell; for both cosolvents, this scenario should result in the grey data points in Figure 4D, which is otherwise simply an expansion of the low cosolvent content-range in Figure 4C. At higher cosolvent content, the Cl^−^ coordination shell would be saturated by cosolvent molecules within this hypothetical strong-attraction model, and this illustrative model becomes less useful. Interestingly, the average values of Cl^−^-water *n*_ClO_ obtained from our simulations are *higher* than the model predictions at *x*_W_ = 0.125 and 0.25, which points to a significant tendency of water molecules to insert between two neighboring Cl^−^ anions, i.e., acting as H-bonded bridges. This is not possible in the corresponding methanol mixtures, as methanol can donate a single H-bond only. The concept of such “bridging” arrangements has been explored in the pure ethaline DES (Kaur et al., 2020), in which Cl^−^ anions have been described as bridges connecting Ch^+^ and EG, rapidly dissolving upon the addition of water.

**FIGURE 5:**
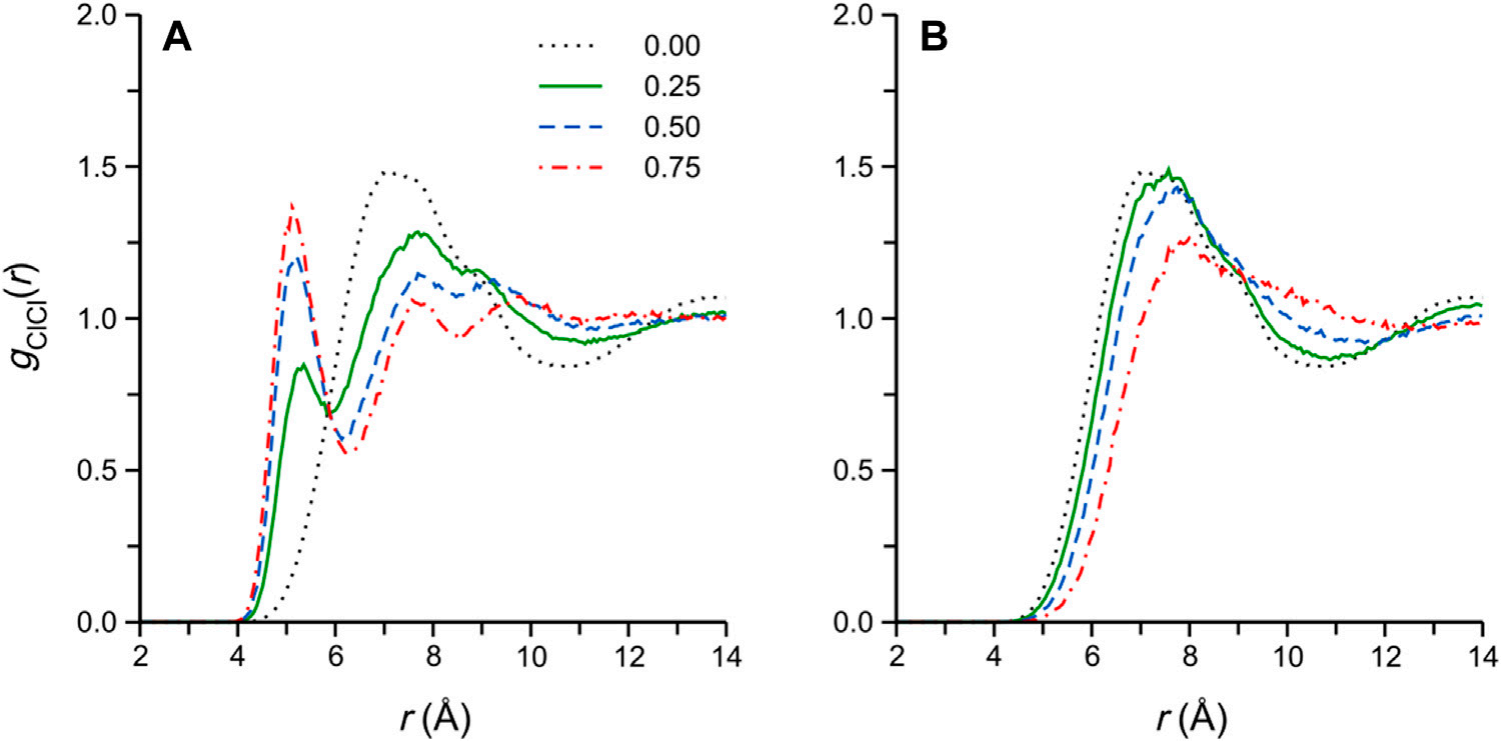
Cl^−^-Cl^-^ RDFs of selected **(A)** DES/water and **(B)** DES/methanol simulated systems. The numbers in the legend in **(A)** refer to the cosolvent mole fraction, and applies to both panels.

The Cl^−^-Cl^−^ RDFs of selected simulated DES/water and methanol mixtures are reported in Figures 5A,B, respectively. These functions are frequently reported for the (simulated) pure DES and its aqueous mixtures (Perkins et al., 2014; Ferreira et al., 2016; Celebi et al., 2019; Kaur et al., 2019), though not for the DES/methanol mixtures (Cea-Klapp et al., 2022). In the pure ethaline DES, the function is characterized by a broad, apparently convoluted, first maximum at *r*_ClCl_ ≈ 7-8 Å (we note that the corresponding RDF reported by Kaur et al. (Kaur et al., 2019) is somewhat different, displaying a clear shoulder at short distances at 303 K); this convoluted first-maximum structure likely arises from Cl^−^ pairs located in the different characteristic “bridging” configurations in the DES (Kaur et al., 2020), i.e., either separated by an EG molecule or Ch^+^, both of which are conformationally flexible.

Turning to the DES/water and methanol mixtures, we note that the changes in, and differences among, the *g*_ClCl_(*r*) in Figure 5 are intriguing, showing clear qualitative differences between the two cosolvents. In the DES/water systems (Figure 5A), a well-defined *g*_ClCl_(*r*) local maximum appears upon the addition of water, first as a low-*r*_ClCl_ shoulder around *r*_ClCl_ ≈ 5.5 Å (at *x*_W_ = 0.125), which then separates and sharpens, gradually shifting to *r*_ClCl_ = 5.0 Å (at *x*_W_ = 0.875) with increasing water content. The appearance of this new *g*_ClCl_(*r*) peak in ethaline/water mixtures was also reported in previous classical simulation studies (Celebi et al., 2019; Kaur et al., 2020) and, interestingly, in a recent *ab initio* MD report by Kirchner and coworkers (Alizadeh et al., 2020), though it appears its significance has not yet received detailed consideration. Indeed, this short-range *g*_ClCl_(*r*) feature is *completely absent* for the corresponding DES/methanol simulated systems (Figure 5B), where the broad first maximum of the pure DES simply loses definition and gradually shifts to larger *r*_ClCl_ with increasing methanol content, as may be expected from the dilution of the DES components.

The RDFs in Figures 3A–D may be similarly integrated to obtain H-bonded coordination numbers for other DES components to Cl^−^. The Cl^−^-Ch^+^ and Cl^−^-EG coordination numbers reported in Supplementary Figure S6, for example, show that, on average, water molecules displace a larger fraction of H-bonded DES components from the Cl^−^ coordination shell compared to methanol at all DES/cosolvent compositions. This finding further underscores the strength of Cl^−^-water ionic H-bonds in the DES/water mixtures.

#### 3.2.2 Simulation configurations and cluster analysis

**FIGURE 6:**
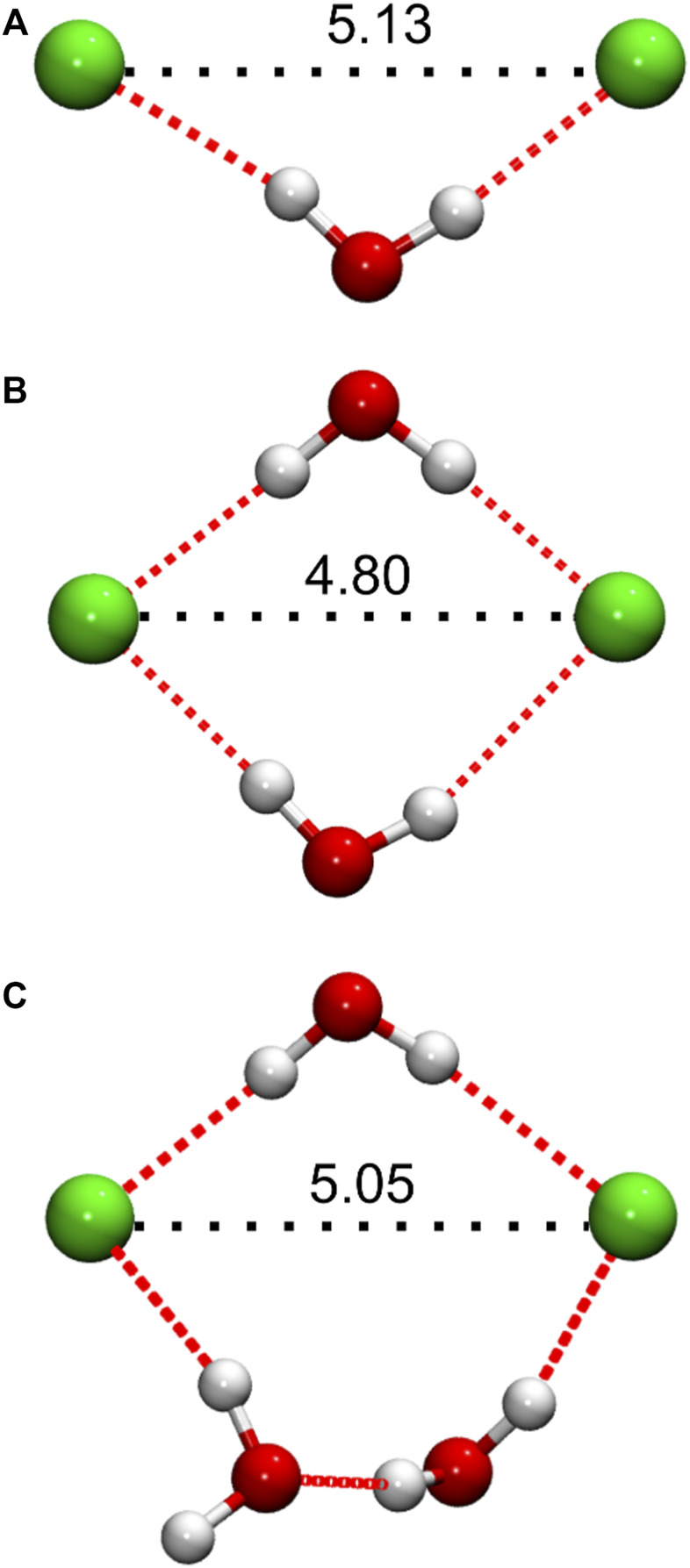
Representative Cl^−^ anion pair configurations from simulated DES/water systems, showing water molecules involved in the formation of H-bonded (dotted red lines) bridging structures, as discussed in the main text. Distances (Å) between the Cl^−^ anions are indicated in black, as relevant to the interpretation of the corresponding *g*_ClCl_(*r*) functions. **(A)** Single, and **(B,C)** double H-bonded bridging arrangements. Configurations **(A,B)** were taken from the DES/water mixture with *x*_W_ = 0.125, while **(C)** was taken from *x*_W_ = 0.5.

The short-range *g*_ClCl_(*r*) maximum that appears in the DES/water mixtures implies the formation of geometrically well-defined structures in which Cl^−^ pairs are drawn closer together compared to distances characteristic of the pure DES structure. This observation is also consistent with our conclusion of water H-bonded “bridges” connecting neighboring Cl^−^ anions, inferred above from the consideration of Cl^−^-water coordination numbers (Figure 4D). Indeed, visual inspection of selected DES/water simulation configurations at low water content reveals an abundance of such arrangements, in which Cl^−^ pairs are connected by one, *or two*, H-bonded water molecule “bridges”; representative configurations are shown in Figures 6A,B. A special case of two H-bonded water bridges connecting a Cl^−^ pair is also frequently observed, particularly at higher *x*_W_, in which one of the bridges consists of *two* H-bonded water molecules, as shown in Figure 6C. At low *x*_W_, a cursory investigation of simulation configurations suggests that Cl^−^ pairs connected by a single H-bonded water molecule exhibit generally greater, and more variable, interionic separations (approximately, ∼5 Å < *r*_ClCl_ ≤ 5.8 Å) compared to pairs connected by two water molecules, e.g., Figures 6B,C, for which often *r*_ClCl_ < 5 Å. With increasing water content, Cl^−^ pairs of the latter two types become more prominent, resulting in a decrease in the average *r*_ClCl_ associated with Cl^−^-pair structures, consistent with the gradual shift of the *g*_ClCl_(*r*) first maximum to *r*_ClCl_ with increasing *x*_W_ (Figure 5A).

**FIGURE 7:**
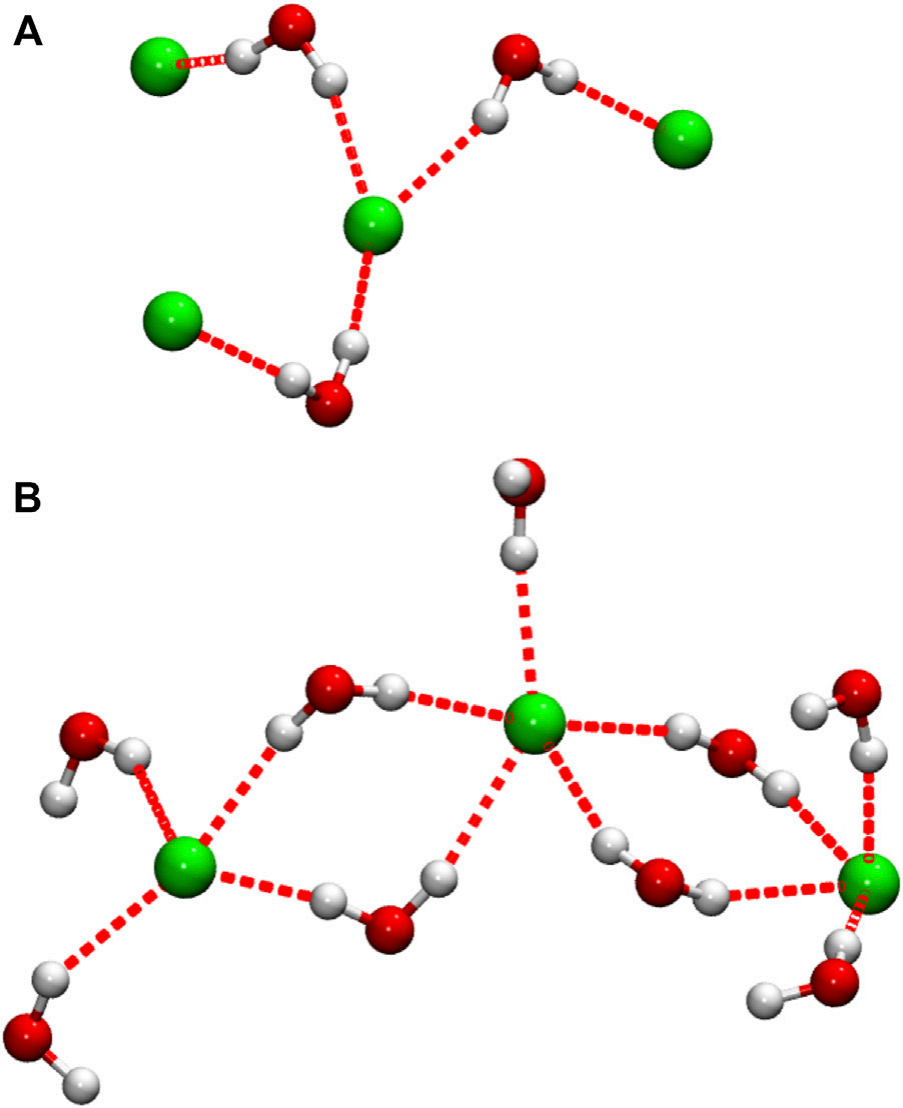
Selected configurations showing Cl^−^ anions connected by H-bonded water bridges. In the interest of clarity, only water molecules directly H-bonded (red dotted lines) to Cl^−^ are shown. Configuration **(A)** was taken from the DES/water mixture at the lowest water content studied (*x*_W_ = 0.125), while **(B)** was taken from the highest water concentration (*x*_W_ = 0.875); configuration **(B)** forms part of a more expansive H-bonded network.

In order to obtain information on the importance of the Cl^−^-pair structures described above, a simple cluster analysis was performed, in which any two anions separated by *r*_ClCl_ ≤ 5.1 Å are considered as the members of the same water-bridged Cl^−^ “cluster”; the chosen cutoff distance corresponds to the position of the closest *g*_ClCl_(*r*) maximum observed in our simulations, at *x*_W_ = 0.875, and is also a distance at which the pure DES function has negligible intensity. The analysis shows that at the lowest water content studied (*x*_W_ = 0.125), on average, about 11% of anions occur in close Cl^−^ pairs. This number increases with increasing water content to ∼30% in the equimolar DES/water mixture, indicating an increase in the occurrence of such pairs, after which it again decreases with further dilution to 16% at the highest DES dilution studied (*x*_W_ = 0.875). Interestingly, the cluster analysis also reveals the presence of larger clusters of this type, containing up to ca. 5–7 anions, depending on the particular system, though these larger clusters account for only a very small anion fraction, < 1% for all systems studied. Examples of these intriguing cluster configurations are presented in Figure 7. Recently, Triolo et al. (Triolo et al., 2021) reported the formation of long H-bonded chains involving water-water and water-Cl^−^ connections in their simulations of a ChCl/water natural DES, which suggests that water-Cl^-^ H-bonded structures may be a more common feature of ChCl-based DES aqueous mixtures.

The Cl^−^ pair configuration in Figure 6C illustrates another important characteristic of the DES/water system, namely a tendency of water molecules to self-associate *via* H-bonding. In fact, in this particular configuration, each water molecule shown is engaged in at least one H-bond with a surrounding water molecule (not shown). The self-association of water molecules in the DES/water mixtures is further exemplified by the full Cl^−^-pair coordination environment configuration shown in Supplementary Figure S7. Similarly, larger water-bridged Cl^−^ configurations, e.g., those shown in Figure 7, typically form part of more extended H-bonded water networks, which also frequently include more distant Cl^−^ anions (i.e., separated by two or more H-bonded water molecules from nearest Cl^−^ neighbors). The prominence of such extended H-bonded networks increases with increasing water content. Interestingly, the question of cosolvent self-association in DES/cosolvent mixtures has not yet received the same computational consideration as IL/water mixtures, for which the phenomenon has been extensively studied by computer simulation techniques (see, e.g. (Jiang et al., 2007), and the review articles (Bhargava et al., 2011; Ma et al., 2018), and references therein). In DESs, however, the situation is complicated considerably by the presence of the neutral HBD; in fact, EG has been reported to self-associate at higher water content in ethaline/water mixtures (Kaur et al., 2020). In their recent report on ethaline/methanol mixture simulations, Cea-Klapp et al. (Cea-Klapp et al., 2022) presented results on the self-association of mixture components, including methanol and EG; these alcohols, having similar H-bonding capabilities, are expected to be more freely miscible in the DES/methanol mixtures (compared to water, that can form H-bonded networks), though it may be interesting to perform an analysis of neutral aggregates containing either species. The extent of cosolvent (or, indeed, DES component) self-association was not studied in the present work, but presents an interesting topic for future detailed study. Moreover, knowledge of cosolvent self-association, or of other DES/cosolvent mixture components, may also contribute to understanding the excess thermodynamic properties of such mixtures, as outlined below.

### 3.3 Interpretation of excess molar enthalpies in context of structural results

The principal structural features of the simulated DES/water mixtures, namely, 1) water molecules interact strongly with DES Cl^−^ anions through ionic H-bonds, acting as H-bonded bridges connecting neighboring anions, and 2) water molecules tend to retain some of the pure water H-bonded network through self-association in the DES/water mixture, are consistent with their strongly exothermic mixing (Ma et al., 2017). Notably, these two structural features appear to be connected, in that Cl^−^ are incorporated into water H-bonded networks, as also recently reported for a ChCl/water natural DES (Triolo et al., 2021).

In the corresponding DES/methanol mixtures, the cosolvent also enters the Cl^−^ coordination shell, forming ionic H-bonds, partially displacing DES components Ch^+^ and EG. The latter, in particular, has been shown to engage in strong ionic H-bonding interactions with Cl^−^ in the pure ethaline DES (Kaur et al., 2019; Alizadeh et al., 2020), either by adopting a *gauche* conformation and coordinating the anion in a bidentate fashion, or by acting as an H-bonded bridge between neighboring anions (or between anions and cations). While methanol, like EG, can form ionic H-bonds with Cl^−^, it cannot facilitate the H-bonded bridging structures that are present in the pure DES, and its displacement of EG from the Cl^−^ coordination shell leads to a reduction of the DES H-bonded network (Cea-Klapp et al., 2022). This structural change is evident in the loss of definition and gradual shift to longer distances of the *g*_ClCl_(*r*) first maximum with increasing methanol content, and also explains the slightly positive *H* ^E^ reported for these mixtures (Wang Y. et al., 2020).

The recent computational-theoretical study of Cea-Klapp et al. (Cea-Klapp et al., 2022) reports that methanol self-association does occur in ethaline/methanol mixtures; while the present study did not investigate this phenomenon, we find it unlikely that the favorable H-bonded ring or even chain structures that have been shown to characterize pure liquid methanol structure should persist in the DES mixtures (Allison et al., 2005), which would make a further positive contribution to *H* ^E^.

## 4 Conclusion

Computer simulation studies of ChCl-based DES/cosolvent mixtures (mainly aqueous mixtures) are increasingly reported, largely focusing on understanding their complex H-bonding interactions; to the best of our knowledge, none have attempted to compute or rationalize excess molar enthalpies. In view of the lack of an established protocol, we tested two related approaches for computing the equivalent excess molar enthalpies for classical MD simulations of mixtures of the DES ethaline (a 1:2 molar ratio mixture of ChCl and ethylene glycol) with either water or methanol. Both approaches reproduce the striking experimental sign difference and general composition dependent variation of the property for the two mixture series (negative at all compositions for aqueous mixtures; positive for methanol mixtures) (Wang Y. et al., 2020).

Structural analyses were aimed at characterizing the DES Cl^−^ anion coordination environment, since strong ionic H-bonding interactions involving this anion are thought to play a central role in determining the excess thermodynamic properties of DES mixtures (Gao et al., 2018). For the aqueous DES mixtures, we showed the curious decrease in closest Cl^−^-Cl^-^ interionic distances with increasing water content—also previously reported in classical (Celebi et al., 2019; Kaur et al., 2020) and *ab initio* MD studies (Alizadeh et al., 2020) of this and related DESs—to be due to water molecules inserting between neighboring Cl^−^ anions, where they form ionic H-bonded bridges that draw the anions closer. The resulting structures are characterized by notably short (≤5 Å) interionic separations. A cluster analysis aimed at identifying such “water-bridged” Cl^−^ pair and larger structures revealed that these account for a notable anion fraction, reaching a maximum of 30% in the equimolar DES/water system and decreasing with further dilution to 16% (water mole fraction *x*_W_ = 0.875). Intriguing larger water-bridged Cl^−^ arrangements, both linear and branched, were also identified; this finding prompts future experimental studies to determine whether these are present in real samples, as their presence may have important implications for applications of the mixtures as solvents or reaction media.

While both water and methanol may be used as cosolvents for reducing the high bulk viscosities of DESs, different intermolecular interactions in the resulting DES/cosolvent - experimentally detectable by measurement of the excess molar enthalpy - may favor one the other for a particular application. We showed that classical MD simulations, that were never used before to calculate *H* ^E^ for DES/cosolvent mixtures, can reproduce the sign and general trend of *H* ^E^ for mixtures these highly complex “pseudo-binary” DES/cosolvent mixtures. Further studies on related systems, will be required to confirm the general applicability of the approach. In addition, considering the very recent development of polarizable force field for this DES (de Souza et al., 2021; Goloviznina et al., 2021), it would be interesting to compare their performance for the calculation of thermodynamical properties.

## Data Availability

The raw data supporting the conclusion of this article will be made available by the authors, without undue reservation.
