# Supplementary material for: MD simulations explain the excess molar enthalpies in pseudo-binary mixtures of a choline chloride-based deep eutectic solvent with water or methanol
Source: Front Chem. 2022 Nov 14;10:983281. doi: 10.3389/fchem.2022.983281 (PMC9702525; doi:10.3389/fchem.2022.983281)
Supplement: Supplementary file 1 [file DataSheet1.DOCX]

Supplementary Material

# Supplementary Data

## Simulation Equilibration Procedure

System equilibration was achieved through a combination of energy minimization and MD simulations with the Amber 16 package (Case et al., 2016). Steepest-descent energy minimization, first with electrostatic interactions turned off (1000 steps) and then on (10,000 steps), were followed by a 1 ns constant volume (NVT) simulation at T = 400 K using the default Berendsen (Berendsen et al., 1984) weak-coupling thermostat (coupling time 1 ps); the simulation time step was Δ*t* = 0.5 fs. A series of NPT simulations (1 ns each, default Berendsen (Berendsen et al., 1984) barostat, *P* = 0.98 bar, with a 1 ps pressure relaxation time) were performed at *T* = 400 K (Δ*t* = 0.5 fs), then at 350 K (Δ*t* = 1 fs), and then at the target temperature of 308 K (Δ*t* = 2 fs), resulting in a stepwise system temperature decrease. Finally, a 1 ns NPT simulation (Δ*t* = 2 fs) was performed at the target temperature with Langevin dynamics temperature regulation, time constant 5 ps^-1^. Other simulation parameters were as described in the main text. For the pure ChCl/ethylene glycol (1:2 molar ratio; “ethaline”) DES, an additional *T* = 298 K equilibration simulation phase was performed (first with the default Berendsen and then Langevin dynamics temperature regulation), followed by a 20 ns production simulation (Langevin dynamics), the average density and structural features of which were compared with the results of Perkins et al. (Perkins et al., 2014).

# Supplementary Figures and Tables

## Supplementary Figures

The figures below show the typical variation of system density and potential energy during the equilibration and production phases of our simulations. The example system is the aqueous mixture of the DES (mole fraction water *x*_W_ = 0.125). For the pure DES, an additional equilibration/production step was performed at 298 K for purposes of validation against the data of Perkins et al. (Perkins et al., 2014), as described in the Supplementary Data section above.

**
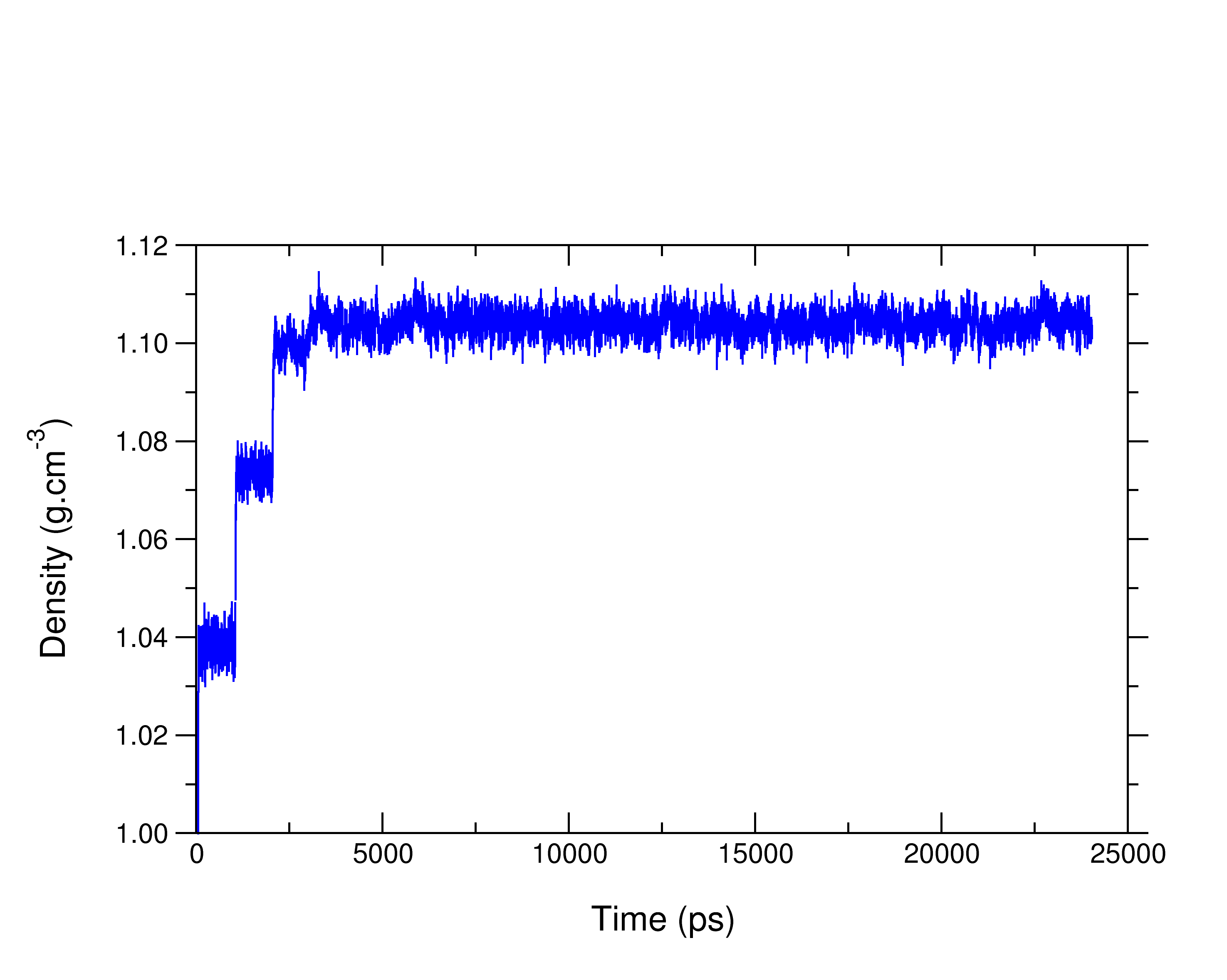
**

Supplementary Figure 1. System density as a function of simulation time over all NPT simulation stages for the DES/water *x*_W_ = 0.125 mixture, both equilibration and production. The stepwise density increase below *t* = 4 ns is due to the equilibration temperature program, 1 ns each at *T* = 400K, then 350 K, and finally 308 K (the target temperature for the production simulation).

**
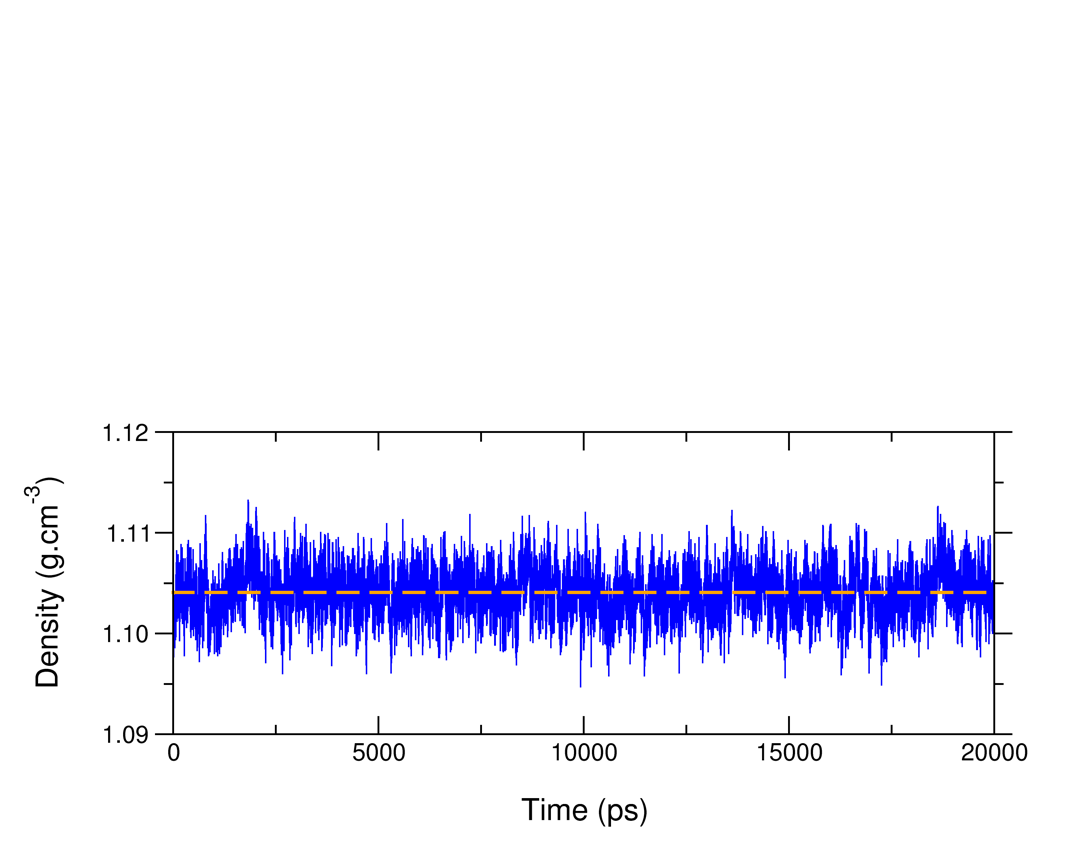
**

Supplementary Figure 2. Density variation of simulated DES/water *x*_W_ = 0.125 mixture during the production stage, i.e., 20 ns Langevin dynamics step at 308 K, 0.98 bar. The dashed orange line shows the average density calculated over the entire production stage data.

**
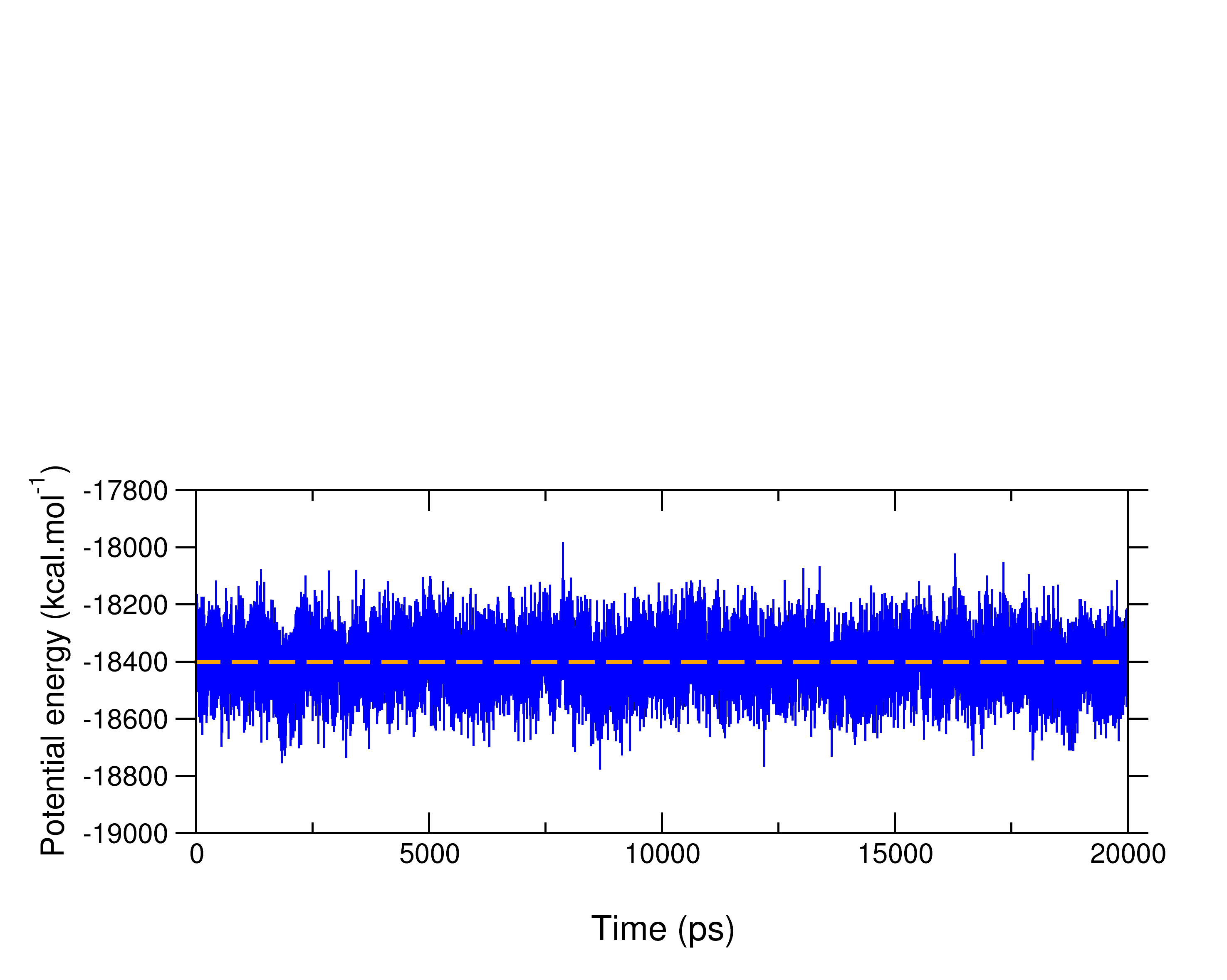
**

Supplementary Figure 3. Potential energy of simulated DES/water *x*_W_ = 0.125 mixture during the production stage, i.e., 20 ns Langevin dynamics step at 308 K, 0.98 bar. The dashed orange line shows the average potential energy calculated over the entire production stage data. Data taken over the final 10 ns of this set were used to calculate the excess molar enthalpy of the system.


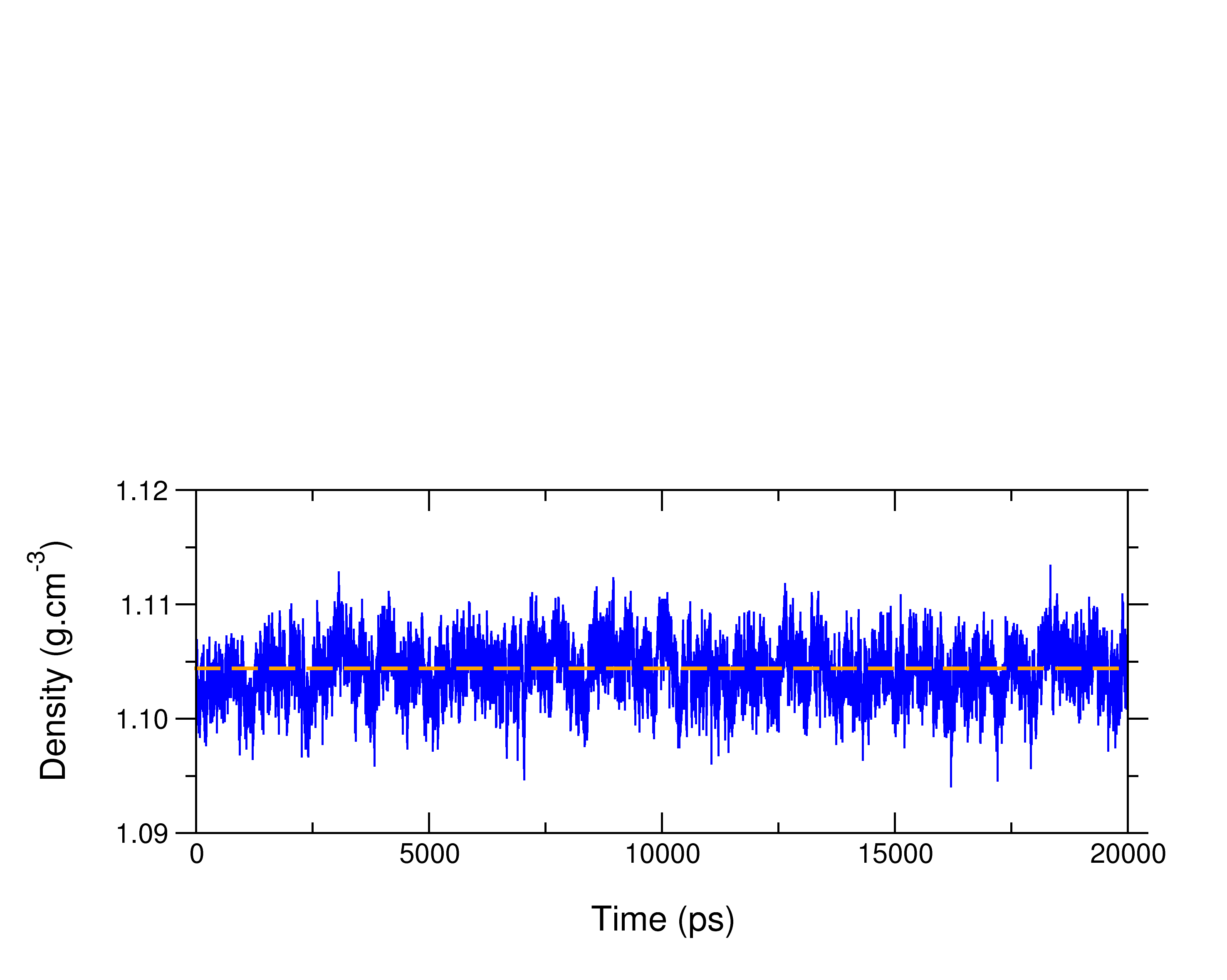


Supplementary Figure 4. Density variation of simulated pure “ethaline” DES during the production stage, i.e., 20 ns Langevin dynamics step at 308 K, 0.98 bar. The dashed orange line shows the average density calculated over the entire production stage data.


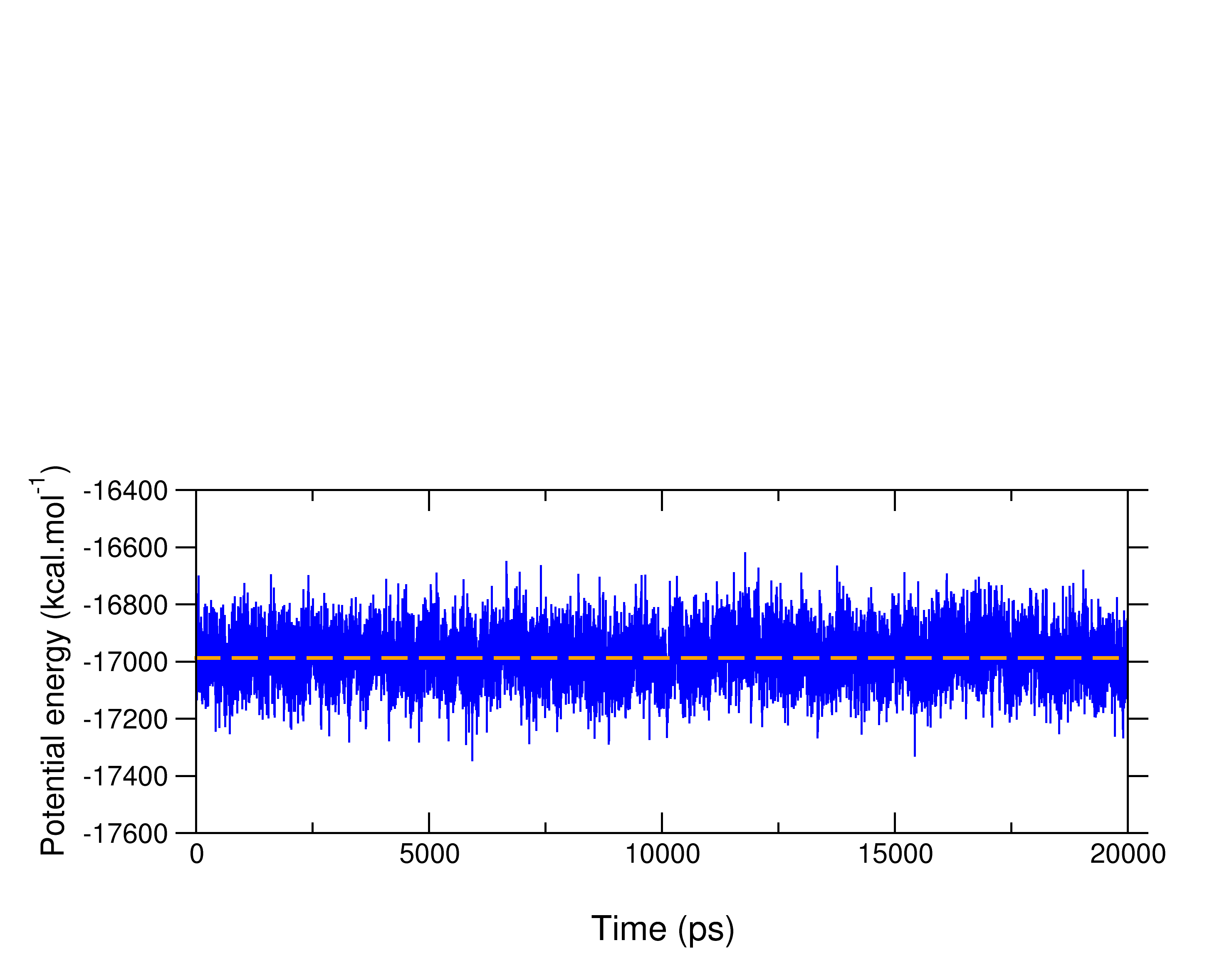


Supplementary Figure 5. Potential energy of simulated pure “ethaline” DES during the production stage, i.e., 20 ns Langevin dynamics step at 308 K, 0.98 bar. The dashed orange line shows the average potential energy calculated over the entire production stage data. Data taken over the final 10 ns of this set were used to calculate the excess molar enthalpy of the system.


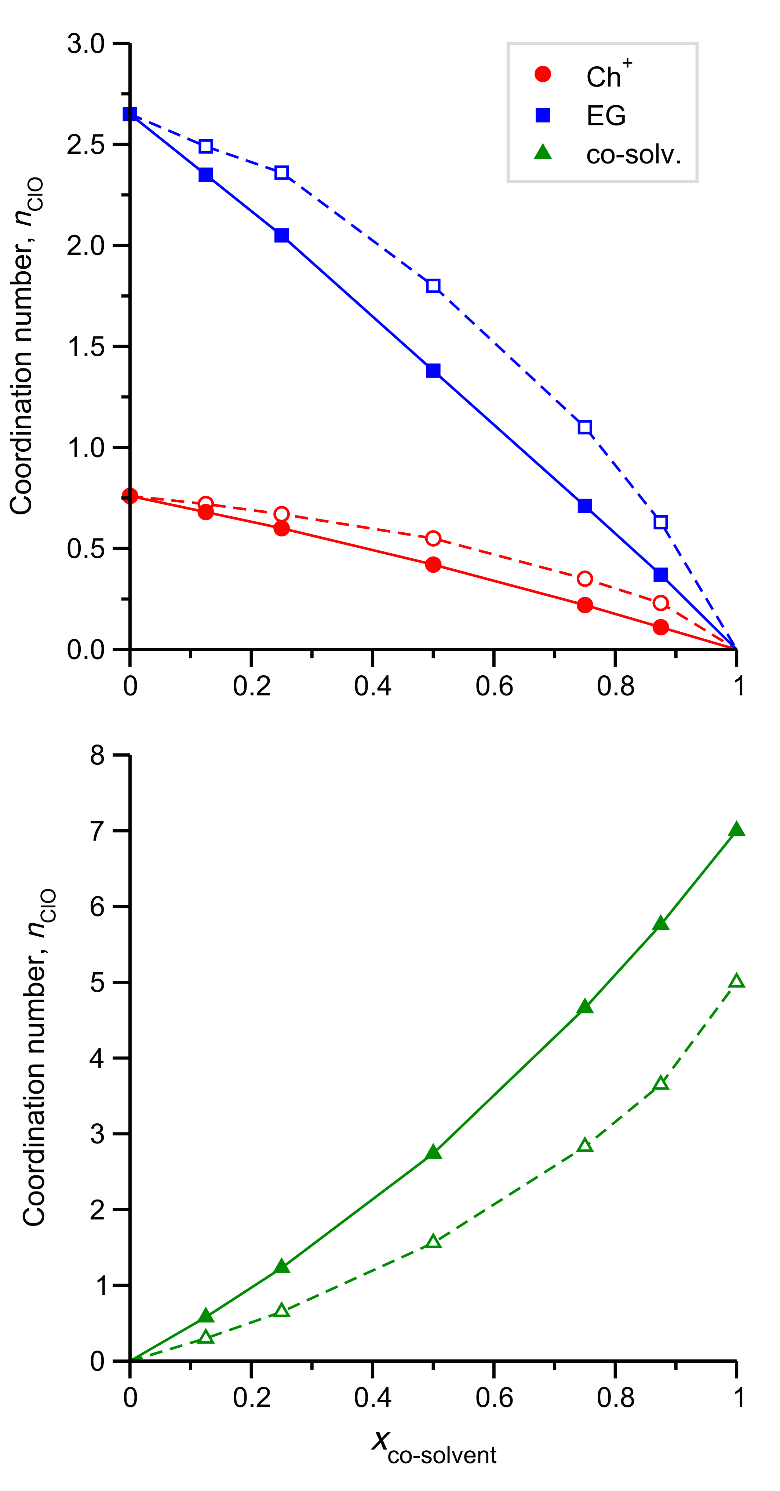


Supplementary Figure 6. Cl^-^-O coordination numbers, *n*_ClO_, obtained by RDF integration, plotted as a function of cosolvent content, *x*_cosolvent_, in simulated ethaline DES/cosolvent mixtures. Data markers are as follows: circles, choline cation (Ch^+^); squares, ethylene glycol (EG); triangles, cosolvent (water or methanol). Solid data markers connected by solid lines pertain to DES/water mixtures, and open markers connected by dashed lines to the corresponding DES/methanol mixtures.


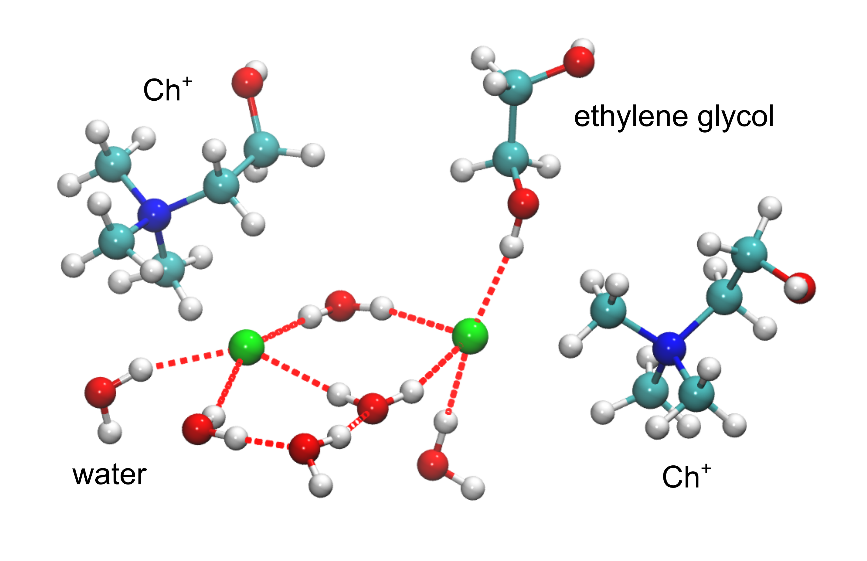


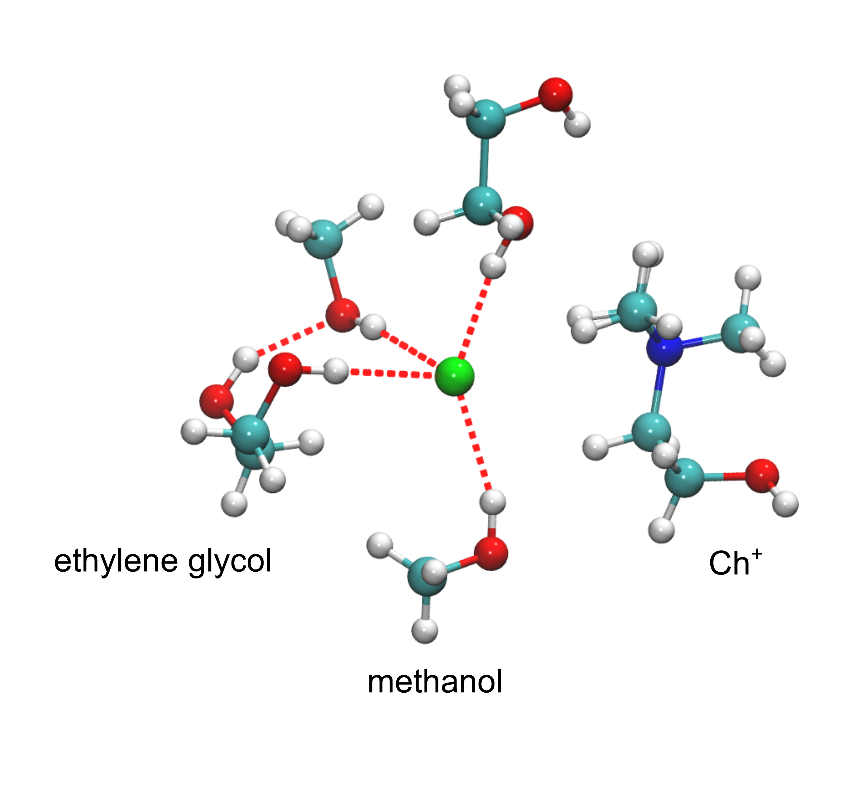


Supplementary Figure 7. Representative Cl^-^ coordination shell configurations from simulations of equimolar DES/cosolvent mixtures: (top) water and (bottom) methanol. Water molecules form strong -O-H⋅⋅⋅Cl^-^ H-bonds between neighboring Cl^-^ anions, resulting in compact water-bridged structures with a characteristic interionic separation that gives rise to the *g*_ClCl_(*r*) first maximum at ~ 5 Å; water molecules also self-associate via strong H-bonding. Methanol molecules are unable to form such H-bonded bridges between Cl^-^.
